# Cord blood metabolomics: a window into future heart health

**DOI:** 10.1038/s41390-025-04530-y

**Published:** 2025-10-20

**Authors:** Anna L. K. Cochrane, Dino A. Giussani

**Affiliations:** 1 Department of Physiology, Development and Neuroscience, University of Cambridge, Cambridge, UK; 2 Loke Centre for Trophoblast Research, University of Cambridge, Cambridge, UK; 3 Strategic Research Initiative in Reproduction, University of Cambridge, Cambridge, UK; 4 Cardiovascular Strategic Research Initiative, University of Cambridge, Cambridge, UK

Cardiometabolic diseases are the leading cause of mortality and morbidity worldwide, with rates of cardiovascular disease, obesity, and diabetes rapidly increasing.^1–3^ This is amplified by an increasing prevalence of multimorbidity of cardiometabolic diseases,^4,5^ with individuals living with obesity at increased risk of developing insulin resistance and hypertension.^6,7^ It is well known that the development of cardiometabolic diseases is underpinned by the interaction between genetics and lifestyle risk factors such as poor diet, lack of exercise, smoking, and air pollution. However, it is increasingly clear that gene-environment interactions before birth may be as, if not more, important in determining future cardiometabolic risk, as described by the Developmental Origins of Health and Disease (DOHaD) hypothesis.^8,9^ As such, offspring exposed to an adverse environment *in utero* have an increased risk of developing cardiometabolic diseases later in life. In humans, the best evidence to support the DOHaD hypothesis comes from studies in women living with obesity who become pregnant before and after undergoing bariatric surgery to induce significant weight loss.^10–12^ Siblings born before surgery were found to have increased cardiometabolic risk, with higher blood pressure, higher adiposity, and poorer glucose homeostasis, compared with their siblings born after surgery.^10–12^ These studies highlight that alterations in the intrauterine environment, even within the same womb, can significantly impact on offspring's future health and disease risk.

The increasing awareness of the importance of early-life environment in determining future cardiometabolic risk has led to a greater focus on prevention rather than treatment to reduce the burden of cardiometabolic disease. To develop successful preventative interventions, it is necessary to identify the underlying mechanisms responsible for the developmental programming of cardiometabolic disease, and to identify as early as possible the individuals at risk. While human epidemiological studies have identified links between early environmental stressors and offspring disease trajectories, pre-clinical animal models have been crucial in identifying causal mechanisms and therapeutic targets.^13–15^ Recently, the availability of ‘omics’ techniques (e.g., transcriptomics, epigenomics, proteomics, and metabolomics), involving high-throughput analysis of complex mixtures of biological molecules with advanced data mining and statistical tools, has provided exciting opportunities for the identification of disease mechanisms, biomarkers, and therapeutic targets.^16–18^ Unbiased omics and multi-omics approaches provide a useful tool for identifying novel pathophysiological pathways in developmental programming, generating hypotheses which can be tested using established pre-clinical animal models. In addition, omics approaches may reveal biomarkers of future disease risk, which can be exploited to identify individuals in need of preventative medicine at critical early stages before disease manifests. The rich datasets created using omics techniques also provide a useful resource that may be exploited by future researchers beyond the remit of the original study, as knowledge in the field progresses.

In this issue of *Pediatric Research*, Fleury et al.^19^ evaluated the human umbilical cord serum metabolome in association with adolescent cardiometabolic risk factors. The study identified 31 metabolic pathways that were dysregulated in the neonatal cord serum associated with increased cardiometabolic disease risk in adolescence, related to glycolysis, gluconeogenesis, and the metabolism of short-chain fatty acids, vitamin C, vitamin B3, glutathione, and amino acids.^19^ Identification of these dysregulated metabolic pathways has the potential to reveal new mechanisms for disease programming and be used to predict future cardiometabolic risk in offspring. While several metabolic pathways identified in this study have previously been associated with pregnancy complications and adverse outcomes, the mechanistic role of these metabolic disturbances in cardiometabolic disease programming remains unclear. Additionally, Fleury et al.^19^ report two distinct cardiometabolic phenotypes that were independently associated with different neonatal serum metabolic signatures. The first group was characterized by higher visceral fat, triglycerides, insulin, leptin, and lower high-density lipoprotein cholesterol; the second group by elevated glucose and systolic blood pressure.^19^ This finding suggests distinct programming mechanisms underpin different aspects of the cardiometabolic disease phenotype, which may arise from common or independent intrauterine stressors.

Previous studies have similarly identified distinct developmental programming pathways for different aspects of the cardiometabolic phenotype in offspring exposed to an adverse environment during pregnancy. For example, studies in humans and mice revealed metabolic dysfunction and cardiac contractile dysfunction in offspring of obese pregnancy that were found to be mediated via one mechanistic pathway and rescued by exercise intervention, while offspring hypertension was mediated by a separate pathway that was not rescued by maternal exercise intervention.^20–22^ Conversely, distinct offspring cardiometabolic phenotypes may be due to exposure to different adverse intrauterine conditions. For example, previous studies reported an association between maternal hypertensive disorders of pregnancy and increased offspring systolic blood pressure only,^23^ while maternal glucose intolerance/gestational diabetes during pregnancy was associated with a broader offspring cardiometabolic phenotype, including higher adiposity, blood pressure, and insulin resistance in male offspring, and dyslipidaemia in female offspring.^24^

**Fig. 1:**
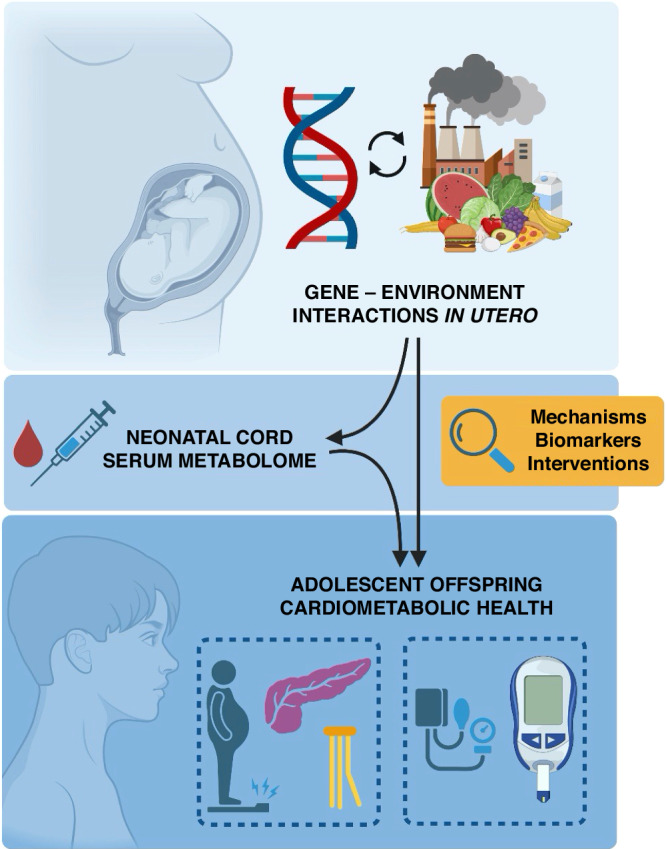
Umbilical cord serum metabolomics provides insights into disease mechanisms underpinning the developmental programming of distinct offspring cardiometabolic phenotypes by adverse intrauterine conditions, facilitating the identification of biomarkers for and interventions against future cardiometabolic risk. Created with BioRender.

The identification of distinct cardiometabolic phenotypes in offspring of adverse pregnancy highlights the need to identify causal links before attributing predictive or mechanistic insight to a metabolic feature or pathway. Therefore, combining associative, unbiased omics studies, such as the one reported by Fleury et al.,^19^ with experiments in established pre-clinical animal models will provide a more complete understanding of programming mechanisms, helping to identify the most suitable therapeutic targets and intervention strategies. A broad, large-scale, unbiased omics approach in cord blood in offspring exposed to adverse intrauterine conditions will certainly expedite movement towards more precise preventative medicine (Fig. 1). Such strategies will help provide an early window into future heart health, the generation of early biomarkers of future disease risk and the prevention of the developmental programming of cardiometabolic disease in offspring of complicated pregnancies.
